# Development of a universal RT-PCR assay for grapevine vitiviruses

**DOI:** 10.1371/journal.pone.0239522

**Published:** 2020-09-22

**Authors:** Alfredo Diaz-Lara, Teresa M. Erickson, Deborah Golino, Maher Al Rwahnih

**Affiliations:** 1 Department of Plant Pathology, University of California-Davis, Davis, California, United States of America; 2 Foundation Plant Services, University of California-Davis, Davis, California, United States of America; Washington State University, UNITED STATES

## Abstract

The genus *Vitivirus* in the family *Betaflexiviridae* includes eleven viruses known to infect grapevine: grapevine vitiviruses A, B, D, E, F, G, H, I, J, L and M (GVA-GVM). Three of these viruses, GVA, GVB and GVD, have been associated with the etiology of rugose wood disease in grapevine and cause agronomically significant losses. The other vitiviruses were more recently discovered and their effects on grapevine are undetermined. To certify grape material for propagation as virus tested, an updated reverse transcription PCR (RT-PCR) assay to detect all known vitiviruses is desirable. To accomplish this, multiple grapevine vitivirus sequences were aligned at the amino acid level to search for conserved motifs. Two highly conserved motifs were found at an ideal distance for RT-PCR detection in the RNA-dependent RNA polymerase region of the replicase protein. The amino acid motifs were back translated to create degenerate primers and used to successfully amplify all eleven grapevine vitiviruses. The RT-PCR primers were used to test a panel of vitivirus-infected vines for inclusivity as well as vines infected with closely related viruses in the *Betaflexiviridae* family (i.e. grapevine pinot gris virus and grapevine rupestris stem pitting-associated virus) for exclusivity. Broader use of these primers to detect vitiviruses in other plant hosts was investigated. In summary, an end-point RT-PCR assay that detects all the known grapevine vitiviruses and potentially other members of the genus *Vitivirus* has been developed. The universal assay represents an alternative to individual assays to reduce the work associated with the diagnosis of vitiviruses, including for regulatory purposes.

## Introduction

The genus *Vitivirus* (family *Betaflexiviridae*) was created in 1997 for the classification of type member grapevine virus A (GVA), a plant virus discovered in grapevine with a filamentous flexuous particle differing from trichoviruses (genus *Trichovirus*) in its genomic arrangement [1]. Vitiviruses have a single-stranded (+) RNA genome encoding five genes: replicase (REP), movement protein, coat protein (CP), nucleic-acid-binding protein and a 20 kDa protein of unknown function. In the 2018 International Committee of Virus Taxonomy Master Species List (https://talk.ictvonline.org/taxonomy/), nine species of vitivirus infecting grapevine are recognized: *Grapevine virus A*, *Grapevine virus B*, *Grapevine virus D*, *Grapevine virus E*, *Grapevine virus F*, *Grapevine virus G*, *Grapevine virus H*, *Grapevine virus I* and *Grapevine virus J*. Since 2019, two more proposed vitiviruses were discovered in grapevine. Grapevine virus L (GVL) was initially identified in RNAseq data and later detected in multiple plants in Croatia, New Zealand and the United States [2]. Grapevine virus M (GVM) was also discovered by high throughput sequencing (HTS) in an American hybrid grapevine [3].

Three different vitiviruses have been associated with the etiology of rugose wood disease in grapevine, a disease with world-wide distribution [4]. GVA is associated with stem grooving on the variety Kober 5BB [5], grapevine virus B was identified as the causal agent of corky bark in the variety LN33 [6], and grapevine virus D was implicated in growth reduction in the rootstock Freedom [7]. Additionally, these vitiviruses are frequently detected in coinfection with grapevine leafroll viruses, resulting in synergistic interactions that can lead to lethal effects in several scion and rootstock combinations [8]. The potential pathogenic role of the remaining grapevine vitiviruses, including proposed members, is still unknown.

Reliable diagnostic methods are critical in determining the viral infection status of a grapevine. Multiple tests are available for the detection of vitiviruses, including biological indexing, real-time or end-point reverse transcription PCR (RT-PCR) and HTS. Biological indicators do not show symptoms for all vitiviruses infecting grape, and RT-PCR assays can fail to detect vitivirus variants containing nucleotide differences at critical primer binding locations. HTS is the most effective means of detecting all vitiviruses but its high cost at large scale limits its use as a screening tool. HTS data is helpful to inform and update RT-PCR primer design as new virus strains are continually being characterized.

In this study, a universal end-point RT-PCR assay involving degenerate primers with the capacity of detecting all the known grapevine vitiviruses was developed. To validate the new assay, eleven grapevines each infected with one of the vitiviruses (i.e. GVA to GVM) were tested. Moreover, a field survey was conducted of known vitivirus-infected grapevines. Following the first reports of vitiviruses in grapevine, several vitiviruses have been discovered in other hosts [9]; consequently, we investigated if the universal assay can detect these vitiviruses.

## Materials and method

### Assay design

With the aim of designing a generic RT-PCR assay for all the known grapevine vitiviruses, all the currently available complete and near-complete genome sequences in GenBank were analyzed. Likewise, available amino acid (aa) sequences for the REP and CP of different grapevine vitiviruses were obtained from GenBank. Thus, similar sequences (i.e. complete genomes, REPs or CPs) were aligned using MUSCLE [10], and Geneious [11] was used to identify a conserved region suitable for assay design. During the REP analysis including sixty-three sequences (accession numbers in S1 Table), multiple highly conserved regions were observed among the alignment and further investigated. Two of these conserved regions, henceforth referred to as motif A (GEFGTFFF) and motif B (PLFCGWR), were back translated using the EMBOSS Backtranambig program [12] for degenerate primer design. This program takes an aa sequence and creates the nucleic acid sequence it could have come from, utilizing nucleotide ambiguity codes to represent all possible codons for each aa. As a result, primers Vitivirus-RdRp-F (5’-GGNGARTTYGGNACNTTYTTYTTY-3’) and Vitivirus-RdRp-R (5’-NCKCCANCCRCARAANARNGG-3’) were generated, which amplify a 219-bp product.

### Plant material and virus source

To empirically validate the new assay, grapevine material infected by ten different vitiviruses (i.e. GVA to GVL) was obtained from the Foundation Plant Services (FPS, University of California-Davis) pipeline of domestic and foreign introductions. A previous study in this grapevine collection revealed the presence of multiple vitiviruses [13]. In addition, GVM-infected material was received from Texas A&M AgriLife Research and Extension Center, Weslaco, Texas. Consequently, eleven grapevines infected by GVA to GVM were included in the assay validation. Finally, a healthy grapevine and plants carrying grapevine Pinot gris virus (GPGV; genus *Trichovirus*) and grapevine rupestris stem pitting-associated virus (GRSPaV; genus *Foveavirus*) were also analyzed to test for assay exclusivity. Like vitiviruses, trichoviruses and foveaviruses belong to the family *Betaflexiviridae*.

### Total RNA extraction

Following the protocol described by [13], plant material was homogenized in extraction buffer and total RNA was extracted using a MagMax Plant RNA Isolation kit (ThermoFisher Scientific, Waltham, MA). As a quality control measure, the presence of RNA was verified using an 18S rRNA assay [14].

### RT-PCR conditions and sensitivity

The universal assay consists of a two-step RT-PCR using the degenerate primers Vitivirus-RdRp-F and Vitivirus-RdRp-R. For reverse transcription (RT), a 20 ul reaction comprised of 7 μl of total RNA template, 4 μl of Invitrogen 5X First Strand buffer, 3 μl of random hexamer primers (0.1 μg/μl), 2 μl of dithiothreitol (0.1 M), 1 μl of dNTPs (10 mM each), 0.2 μl of RNasin Ribonuclease Inhibitor (40 U/μl) (Promega, Madison, WI), 0.25 μl of SuperScript II Reverse Transcriptase (200 U/μl) (Invitrogen, Carlsbad, CA) and 2.55 μl of nuclease-free water, was incubated at 25°C for 15 min, 42°C for 90 min and 72°C for 15 min. The PCR reaction includes 2 μl of RT product, 5 μl of Promega 5X GoTaq Green buffer, 2 μl of MgCl_2_ (25 mM), 1 μl of dNTPs (10 mM of each), 1 μl each of Vitivirus-RdRp-F and Vitivirus-RdRp-R (50 μM), 0.25 μl of GoTaq G2 Flexi DNA Polymerase (5 U/μl) (Promega, Madison, WI) and 12.75 μl of nuclease-free water. Amplification conditions consist of 3 min at 94°C, 35 cycles of 30 s at 94°C, 45 s at 53°C, 1 min at 72°C, and a final extension step of 7 min at 72°C. PCR products were electrophoresed on a 1.5% agarose gel in TAE buffer containing Gel Red nucleic acid gel stain (Biotium, Hayward, CA).

Assay sensitivity was investigated using serial dilutions (up to 10^−5^) of total RNA from two grapevines infected by GVA and GVB. The universal assay was compared against two commonly used RT-PCR assays specific for either GVA or GVB [15, 16].

### Cloning and sequencing

Amplicons generated by the universal assay were purified using the DNA Clean & Concentrator -5 kit (Zymo Research Corp., Irvine, CA), cloned using the TOPO TA Cloning kit and transformed into One Shot Top 10 chemically competent cells of *Escherichia coli* (Invitrogen, Carlsbad, CA) following the manufacturer’s instructions. Ten clones per PCR transformation were Sanger sequenced employing M13 primers at the University of California-Davis DNA Sequencing Facility.

### Field survey

The Davis Virus Collection (DVC) located at the University of California-Davis contains 385 grapevines representing multiple domestic selections [17]. Plants in this collection are infected by different viruses including vitiviruses. To further investigate the reliability of the universal assay, all the grapevines at DVC were tested, and results compared against previous results obtained by using PCR primers targeting individual vitiviruses [3, 13].

### Assay inclusivity and exclusivity

To determine if the developed RT-PCR assay can detect vitiviruses in other hosts, mint and blueberry material infected by mint virus 2 (MV-2) [18] and blueberry green mosaic-associated virus (BGMaV) [19], respectively, were tested. Additionally, sequences of motif A and B were compared against proteins of viruses within the genus *Vitivirus* (GenBank taxid:184751), genus *Trichovirus* (GenBank taxid:40276) and genus *Foveavirus* (GenBank taxid:129725) using the BLASTp program [20].

## Results

### Identification of conserved sequence motifs

**Fig 1:**
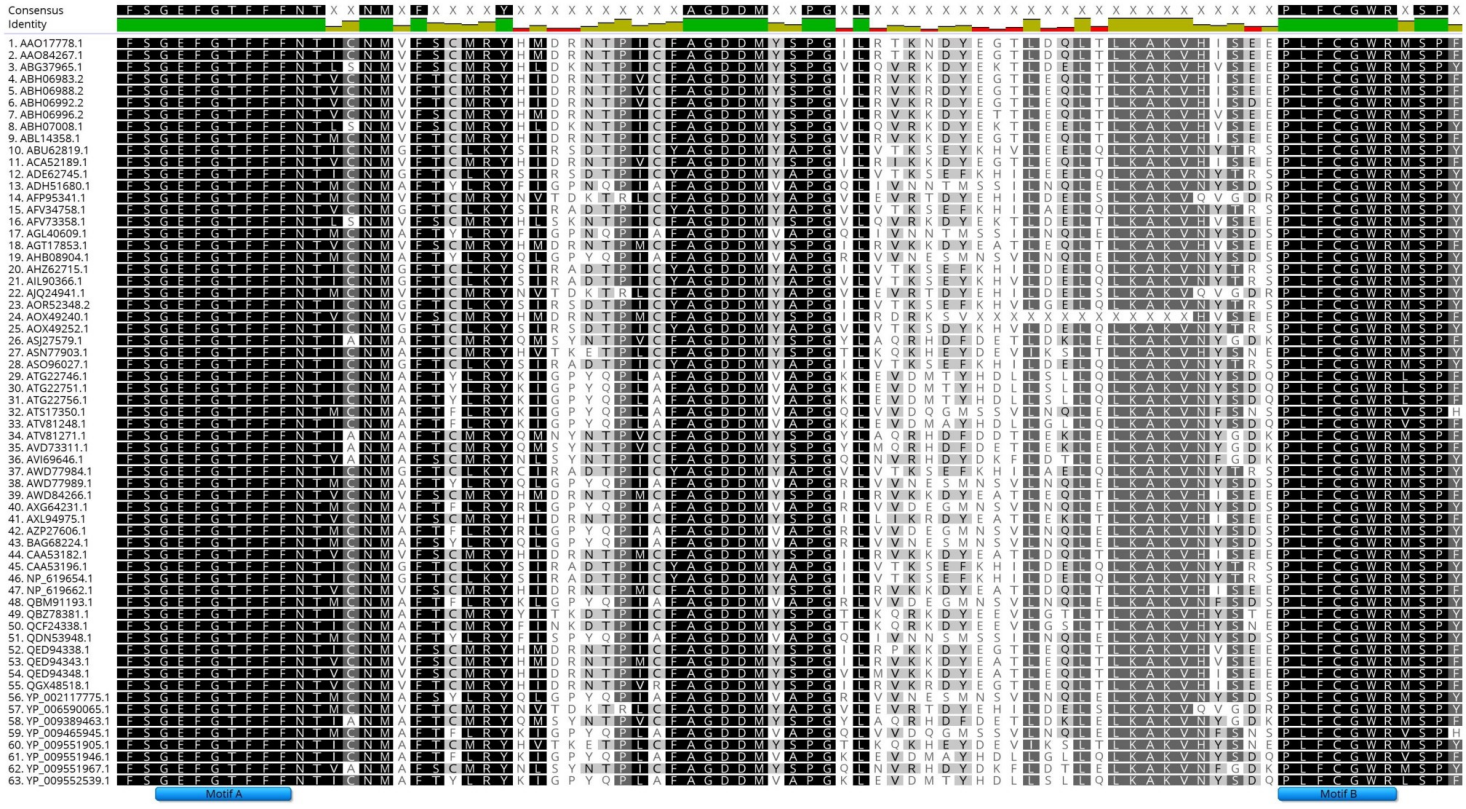
Alignment of replicase proteins present in different grapevine vitiviruses. Multiple amino acid sequences available in GenBank were aligned to identify a conserved region among the viruses (GVA to GVM). Consensus and identity are displayed at the top of the alignment. Left column corresponds to GenBank accession numbers. Identified motifs (A and B) are indicated in blue color.

The search for regions of high homology in vitivirus nucleotide sequences and CP amino acid sequences were unsuccessful. MUSCLE alignment of REP proteins encoded by grapevine vitiviruses, including definitive and tentative species, did show a considerable level of conservation. Two conserved motifs (i.e. motif A and motif B) were identified in the RdRp domain which were long enough to generate PCR primers (Fig 1).

### Validation of universal assay

**Fig 2:**
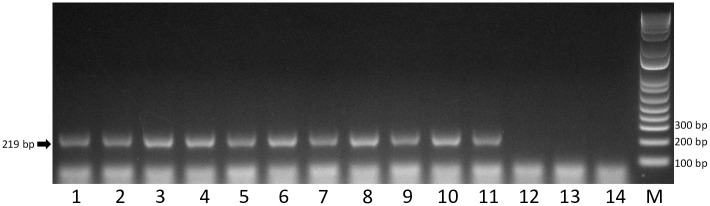
Detection of different grapevine vitiviruses by reverse transcription PCR using degenerate primers. Lane 1, grapevine virus A; lane 2, grapevine virus B; lane 3, grapevine virus D; lane 4, grapevine virus E; lane 5, grapevine virus F; lane 6, grapevine virus G; lane 7, grapevine virus H; lane 8, grapevine virus I; lane 9, grapevine virus J; lane 10, grapevine virus L; lane 11, grapevine virus M; lane 12, grapevine Pinot gris virus; lane 13, grapevine rupestris stem pitting-associated virus; lane 14, healthy grapevine; lane M, 1 Kb Plus DNA Ladder marker. Expected amplicon size: 219 bp.

During the validation of the universal assay using eleven vitivirus-infected grapevines, all tested positive and amplicons of the expected size were observed (Fig 2). The PCR products were cloned, sequenced and confirmed to match the identity of the intended vitivirus (i.e. GVA to GVM). In contrast, the RT-PCR assay was shown not to amplify any detectable product from healthy *Vitis* sp. or GPGV- or GRSPaV-infected grapevines (Fig 2).

**Fig 3:**
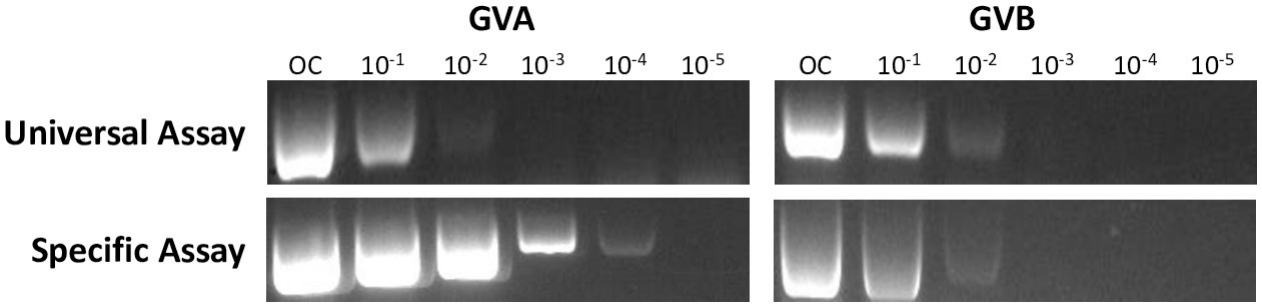
Detection of a dilution series of grapevine viruses A and B (GVA and GVB) by universal and GVA or GVB specific assays. OC, original concentration; total RNA diluted in water from 10^−1^ to 10^−5^.

To investigate the sensitivity of the generic assay, a dilution series of 14 ng/ul total plant RNA was used as a template for RT-PCR. The new assay could detect the presence of GVA and GVB up to a 10^−2^ dilution, amplifying a faint band in both cases (Fig 3). The specific assay for GVA was more sensitive than the universal assay, generating a visible product at a 10^−4^ dilution. The universal assay and the GVB specific assay displayed similar levels of sensitivity (Fig 3).

### Field survey

The universal RT-PCR assay was used to evaluate the occurrence of vitiviruses in the DVC grapevine collection maintained by the University of California-Davis. Independently, the same plants were analyzed by individual assays for GVA to GVM. Grapevines testing positive by the universal assay tested positive by at least one of the individual assays, and all plants testing negative by the universal assay were found free of vitiviruses by all of the individual assays (S2 Table). Overall, 185 of 385 (48%) grapevines at the DVC exhibited a single or mixed infection by vitiviruses GVA, B, D, E and F.

### Assay inclusivity and exclusivity

**Fig 4:**
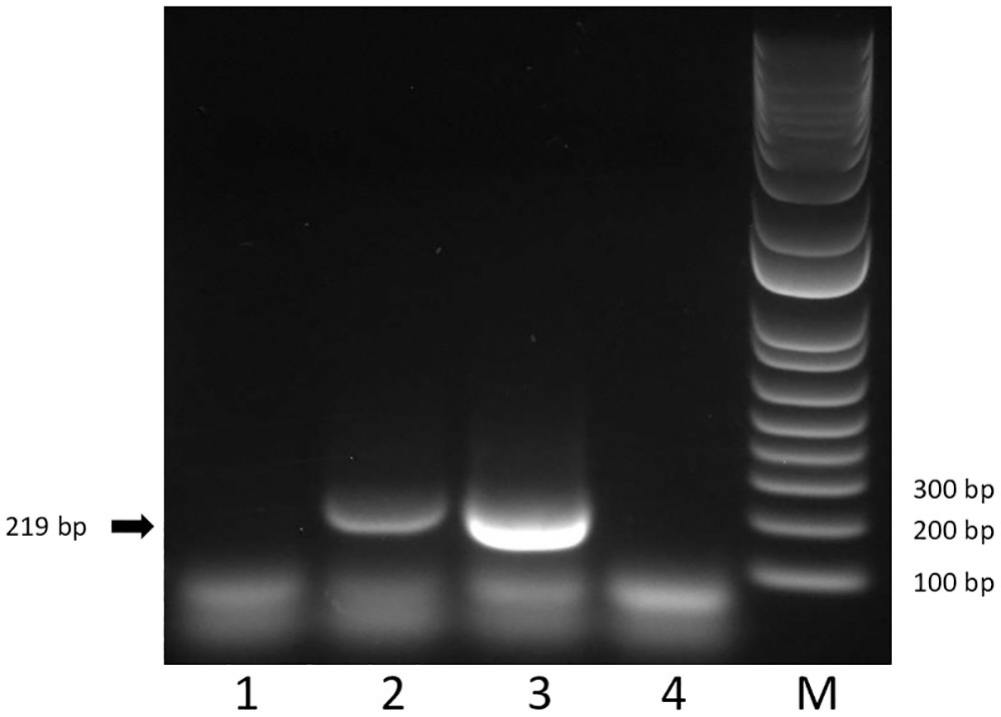
Analysis of different hosts infected by vitiviruses using the universal assay. Lane 1, blueberry infected by blueberry green mosaic-associated virus; lane 2, mint infected by mint virus 2; lane 3, grapevine infected by grapevine virus A; lane 4, healthy grapevine; lane M, 1 Kb Plus DNA Ladder marker.

Given the success of the universal assay to amplify all the known grapevine vitiviruses, we investigated if this assay could also detect vitiviruses infecting other hosts. The universal assay generated a 219-bp product from the MV-2-infected mint that was confirmed by cloning and Sanger sequencing, but failed to amplify a product from the BGMaV-infected blueberry (Fig 4).

Subsequent *in-silico* analysis of motifs A and B in the seven non-grapevine infecting vitiviruses, actinidia virus A and B, blackberry virus A, agave tequilana leaf virus, arracacha virus V (AVV), MV-2 and BGMaV, revealed an 100% identity and coverage for five of these viruses; however, two vitiviruses, BGMaV and AVV had 100% identity and 87% coverage (Table 1). Analysis of BGMaV and AVV sequences indicated a single aa variation in the C-terminus of motif A. Lastly, not a single trichovirus or foveavirus displayed 100% identity and coverage for both motifs (S3 and S4 Tables).

**Table 1:** BLASTp analysis of amino acid (aa) sequences, motif A and motif B, present in different vitiviruses.

Vitivirus	Motif A			Motif B		
	% Identity	% Coverage	aa Mismatch	% Identity	% Coverage	aa Mismatch
Grapevine virus A	100	100	0	100	100	0
Grapevine virus B	100	100	0	100	100	0
Grapevine virus D	100	100	0	100	100	0
Grapevine virus E	100	100	0	100	100	0
Grapevine virus F	100	100	0	100	100	0
Grapevine virus G	100	100	0	100	100	0
Grapevine virus H	100	100	0	100	100	0
Grapevine virus I	100	100	0	100	100	0
Grapevine virus J	100	100	0	100	100	0
Grapevine virus L	100	100	0	100	100	0
Grapevine virus M	100	100	0	100	100	0
Actinidia virus A	100	100	0	100	100	0
Actinidia virus B	100	100	0	100	100	0
Blackberry virus A	100	100	0	100	100	0
Agave tequilana leaf virus	100	100	0	100	100	0
Mint virus 2	100	100	0	100	100	0
Blueberry green mosaic associated virus	100	87	1	100	100	0
Arracacha virus V	100	87	1	100	100	0

## Discussion

A universal assay able to detect all known grapevine vitiviruses and potentially other members of the genus *Vitivirus* was developed here based on sequence data available in GenBank. The presence of highly conserved motifs in the REP protein allowed the design of end-point RT-PCR primers, providing an alternative assay to reduce the work associated with the diagnosis of vitiviruses.

The extensive sequence divergence existing among grapevine vitiviruses, observed at the nucleotide and aa levels, makes it difficult to design a test with broad-range detection. RT-PCR with degenerate primers is a simple strategy that is frequently used for the specific and simultaneous detection of multiple viruses [21]. Assays involving degenerate primers targeting grapevine vitiviruses have been described before [22, 23], however, these studies were conducted in the pre-HTS era, when fewer vitiviruses were known and sequence data was limited.

Although *Vitis* spp. is recognized as the main host associated with the genus *Vitivirus*, vitiviruses have been identified in other perennial hosts, the majority of which are woody plants [9]. For example, vitiviruses have been reported in blackberry, mint, agave and recently in blueberry. The universal assay successfully detected MV-2 in mint, however failed to detect BGMaV in blueberry. Additional investigation revealed a variation in motif A of BGMaV (i.e. a single aa change), and a similar scenario was observed in AVV. Based on our PCR results we predict that the universal assay will miss AVV during diagnosis, though, the rest of the known vitiviruses do not display any aa discrepancy in motifs A or B and they should be detected by the assay. The family *Betaflexiviridae* comprises twelve different genera (https://talk.ictvonline.org/taxonomy/), including *Vitivirus*, *Trichovirus* and *Foveavirus*. After *in-silico* and *in-vitro* analyses of trichoviruses and foveaviruses, we did not find evidence for cross-reaction by the universal assay.

A single test for all known grapevine vitiviruses can be a useful tool for improving efficiency and reducing costs of large-scale surveys. Potentially, this generic assay may detect novel *Vitivirus* species in grapevine and other hosts given its unbiased nature. Similar assays have been developed for carlaviruses [24], nepoviruses [25] and different members of the family *Betaflexiviridae* [26].

Grapevine is clonally propagated, consequently, to prevent the spread of vitiviruses, it is critical to use virus tested material. The assay developed here will be made available to diagnostic labs and will facilitate the production of certified virus-tested propagation material and the effective control of vitiviruses.

## Supporting information

S1 Table Replicase proteins of grapevine vitiviruses included in this study and available in GenBank. (DOCX)

S2 Table Testing results of the field survey, comparison between universal and individual assays. (XLSX)

S3 Table BLASTp analysis of amino acid (aa) sequences, motif A and motif B, present in different trichoviruses. (DOCX)

S4 Table BLASTp analysis of amino acid (aa) sequences, motif A and motif B, present in different foveaviruses. (DOCX)

S1 Raw Images (PDF)
