# Supplementary material for: Development of a universal RT-PCR assay for grapevine vitiviruses
Source: PLoS One. 2020 Sep 22;15(9):e0239522. doi: 10.1371/journal.pone.0239522 (PMC7508359; doi:10.1371/journal.pone.0239522)
Supplement: S1 Table — (DOCX) [file pone.0239522.s001.docx]

**S1 Table.** Replicase proteins of grapevine vitiviruses included in this study and available in GenBank.

| **GenBank Accession** | **Grapevine Vitivirus** |
| --- | --- |
| AAO17778.1 | Grapevine virus A |
| AAO84267.1 | Grapevine virus A |
| ABG37965.1 | Grapevine virus A |
| ABH06983.2 | Grapevine virus A |
| ABH06988.2 | Grapevine virus A |
| ABH06992.2 | Grapevine virus A |
| ABH06996.2 | Grapevine virus A |
| ABH07008.1 | Grapevine virus A |
| ABL14358.1 | Grapevine virus A |
| ACA52189.1 | Grapevine virus A |
| AFV73358.1 | Grapevine virus A |
| AGT17853.1 | Grapevine virus A |
| AOX49240.1 | Grapevine virus A |
| AWD84266.1 | Grapevine virus A |
| AXL94975.1 | Grapevine virus A |
| CAA53182.1 | Grapevine virus A |
| NP_619662.1 | Grapevine virus A |
| QED94338.1 | Grapevine virus A |
| QED94343.1 | Grapevine virus A |
| QED94348.1 | Grapevine virus A |
| QGX48518.1 | Grapevine virus A |
| ABU62819.1 | Grapevine virus B |
| ADE62745.1 | Grapevine virus B |
| AFV34758.1 | Grapevine virus B |
| AHZ62715.1 | Grapevine virus B |
| AIL90366.1 | Grapevine virus B |
| AOR52348.2 | Grapevine virus B |
| AOX49252.1 | Grapevine virus B |
| ASO96027.1 | Grapevine virus B |
| AWD77984.1 | Grapevine virus B |
| CAA53196.1 | Grapevine virus B |
| NP_619654.1 | Grapevine virus B |
| AVD73311.1 | Grapevine virus D |
| ADH51680.1 | Grapevine virus E |
| AGL40609.1 | Grapevine virus E |
| AHB08904.1 | Grapevine virus E |
| AWD77989.1 | Grapevine virus E |
| BAG68224.1 | Grapevine virus E |
| QDN53948.1 | Grapevine virus E |
| YP_002117775.1 | Grapevine virus E |
| AFP95341.1 | Grapevine virus F |
| AJQ24941.1 | Grapevine virus F |
| YP_006590065.1 | Grapevine virus F |
| ATG22746.1 | Grapevine virus G |
| ATG22751.1 | Grapevine virus G |
| ATG22756.1 | Grapevine virus G |
| ATV81248.1 | Grapevine virus G |
| YP_009551946.1 | Grapevine virus G |
| YP_009552539.1 | Grapevine virus G |
| ASN77903.1 | Grapevine virus H |
| QBZ78381.1 | Grapevine virus H |
| YP_009551905.1 | Grapevine virus H |
| ATS17350.1 | Grapevine virus I |
| YP_009465945.1 | Grapevine virus I |
| AVI69646.1 | Grapevine virus J |
| YP_009551967.1 | Grapevine virus J |
| ASJ27579.1 | Grapevine virus K |
| ATV81271.1 | Grapevine virus K |
| YP_009389463.1 | Grapevine virus K |
| AXG64231.1 | Grapevine virus L |
| AZP27606.1 | Grapevine virus L |
| QBM91193.1 | Grapevine virus L |
| QCF24338.1 | Grapevine virus M |
