# Supplementary material for: Development of a universal RT-PCR assay for grapevine vitiviruses
Source: PLoS One. 2020 Sep 22;15(9):e0239522. doi: 10.1371/journal.pone.0239522 (PMC7508359; doi:10.1371/journal.pone.0239522)
Supplement: S3 Table — (DOCX) [file pone.0239522.s003.docx]

**S3 Table.** BLASTp analysis of amino acid (aa) sequences, motif A and motif B, present in different trichoviruses.

| **Trichovirus** | **Motif A** | | **Motif B** | |
| --- | --- | --- | --- | --- |
|  | **% Coverage** | **% Identity** | **% Coverage** | **% Identity** |
| Grapevine berry inner necrosis virus | 63 | 75 | 100 | 100 |
| Grapevine Pinot gris virus | 100 | 75 | 100 | 100 |
| Fig latent virus 1 | 100 | 75 | 100 | 100 |
| Apricot pseudo-chlorotic leaf spot virus | 100 | 75 | 100 | 85 |
| Cherry mottle leaf virus | 100 | 75 | 100 | 85 |
| Apple chlorotic leaf spot virus | 100 | 75 | 100 | 85 |
| Peach mosaic virus | 100 | 75 | 100 | 85 |
| Phlomis mottle virus | 100 | 75 | 85 | 83 |
