# Supplementary material for: Development of a universal RT-PCR assay for grapevine vitiviruses
Source: PLoS One. 2020 Sep 22;15(9):e0239522. doi: 10.1371/journal.pone.0239522 (PMC7508359; doi:10.1371/journal.pone.0239522)
Supplement: S4 Table — (DOCX) [file pone.0239522.s004.docx]

**S4 Table.** BLASTp analysis of amino acid (aa) sequences, motif A and motif B, present in different foveaviruses.

| **Foveavirus** | **Motif A** | | **Motif B** | |
| --- | --- | --- | --- | --- |
|  | **% Coverage** | **% Identity** | **% Coverage** | **% Identity** |
| Peach chlorotic mottle virus | 100 | 72 | 85 | 83 |
| Apple stem pitting virus | 50 | 100 | 85 | 83 |
| Asian prunus virus 1 | 50 | 100 | 85 | 83 |
| Asian prunus virus 2 | 50 | 100 | 85 | 83 |
| Grapevine rupestris stem pitting-associated virus | 100 | 62 | 85 | 83 |
| Maize associated foveavirus | 100 | 62 | 85 | 83 |
| Cherry virus B | 100 | 62 | 85 | 83 |
| Rubus canadensis virus 1 | 100 | 62 | 85 | 83 |
| Grapevine virus T | 100 | 62 | 85 | 83 |
| Apricot latent virus | 100 | 62 | 85 | 83 |
