# Supplementary material for: Development of a universal RT-PCR assay for grapevine vitiviruses
Source: PLoS One. 2020 Sep 22;15(9):e0239522. doi: 10.1371/journal.pone.0239522 (PMC7508359; doi:10.1371/journal.pone.0239522)

Fig 2. Detection of different grapevine vitiviruses by reverse transcription PCR using degenerate primers. Lane 1, grapevine virus A; lane 2, grapevine virus B; lane 3, grapevine virus D; lane 4, grapevine virus E; lane 5, grapevine virus F; lane 6, grapevine virus G; lane 7, grapevine virus H; lane 8, grapevine virus I; lane 9, grapevine virus J; lane 10, grapevine virus L; lane 11, grapevine virus M; lane 12, grapevine Pinot gris virus; lane 13, grapevine rupestris stem pitting-associated virus; lane 14, healthy grapevine; lane M, 1 Kb Plus DNA Ladder marker. Expected amplicon size: 219 bp.

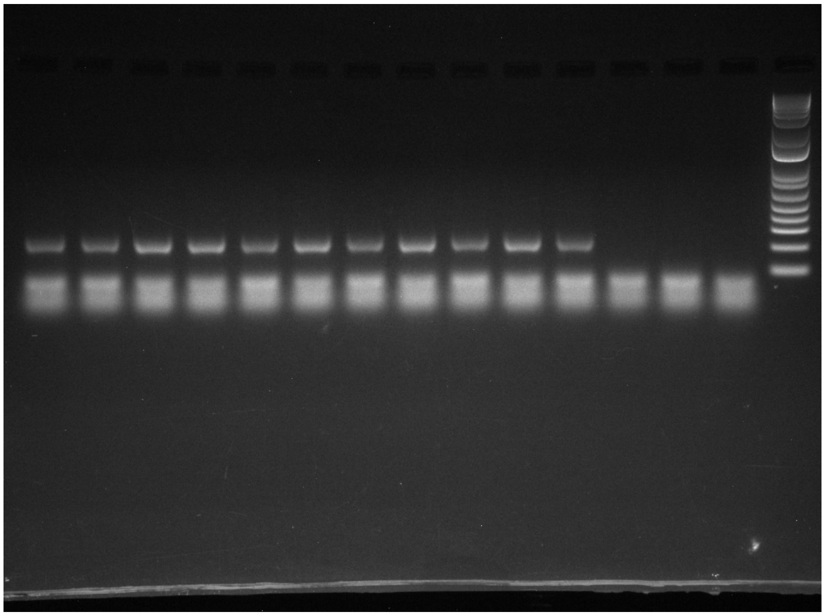

Fig 3. Detection of a dilution series of grapevine viruses A and B (GVA and GVB) by universal and GVA or GVB specific assays. OC, original concentration; total RNA diluted in water from 10^{-1} to 10^{-5}.

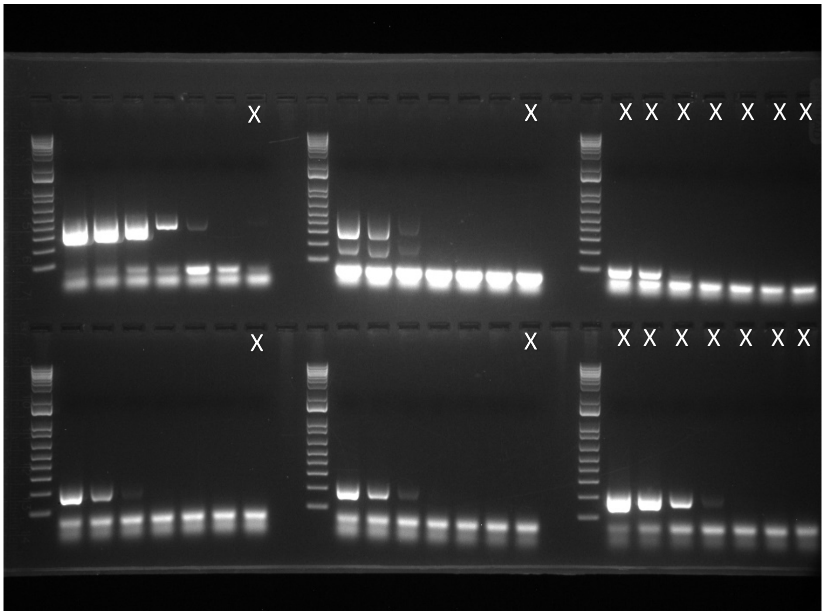

Fig 4. Analysis of different hosts infected by vitiviruses using the universal assay. Lane 1, blueberry infected by blueberry green mosaic-associated virus; lane 2, mint infected by mint virus 2; lane 3, grapevine infected by grapevine virus A; lane 4, healthy grapevine; lane M, 1 Kb Plus DNA Ladder marker.

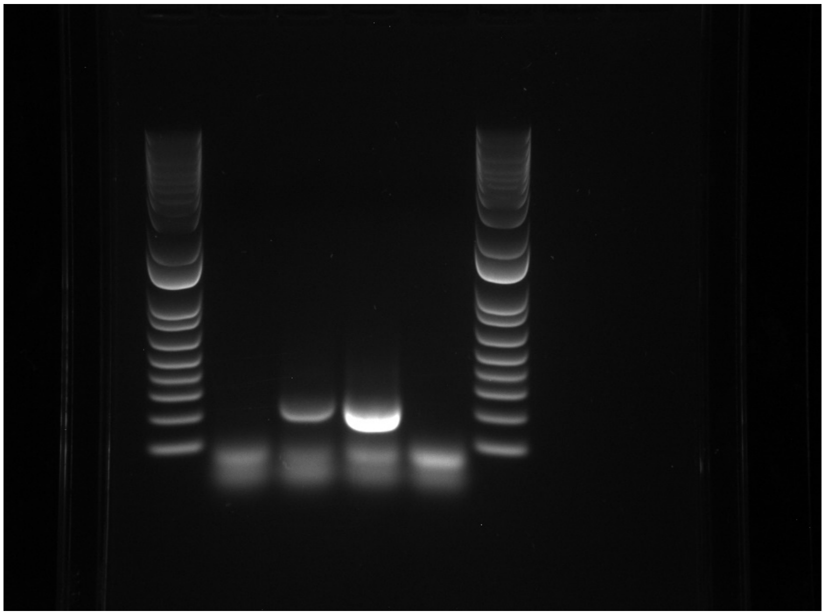

Supplement: S1 Raw Images — (PDF) [file pone.0239522.s005.pdf]
